# Identification of a *Caldariomyces fumago* Mutant Secreting an Inactive Form of Chloroperoxidase Lacking the Heme Group and *N*-Glycans

**DOI:** 10.1371/journal.pone.0067857

**Published:** 2013-07-02

**Authors:** Sonja Hüttmann, Markus Buchhaupt, Jens Schrader

**Affiliations:** DECHEMA Research Institute, Frankfurt am Main, Germany; The University of Texas at San Antonio, United States of America

## Abstract

By mutant colony screening of *Caldariomyces fumago* a mutant was isolated which was slightly greenish on fructose minimal medium and grew slower in comparison to the wild type. The supernatant samples lacked the Soret band typical for the heme group of the CPO and nearly no CPO activity was detected. SDS-PAGE analysis of mutant culture supernatant samples showed production of a 38–40 kDa protein while wild type samples contain the 42 kDa CPO protein. Protein identification using nanoLC-ESI-MS/MS was performed and based on three peptides the protein in the mutant culture was identified as CPO. No differences in the CPO gene sequences of wild type and mutant were found indicating a post-translational defect in protein maturation. Deglycosylation experiments using CPO from wild type and mutant were carried out. After removing N-linked oligosaccharides from wild type CPO a protein band at 38–40 kDa was detected. Our results reveal that the mutant protein lacks the heme group as well as the N-glycans.

## Introduction

Glycosylation is a common post-translational modification of proteins in eukaryotic systems. Many proteins are glycosylated for the purpose of transportation out of the cell and appropriate targeting, for the distribution among different intracellular compartments, for controlling confirmation and folding or to enable adhesion on cell surfaces [1]. Furthermore, glycosylation can stabilize proteins and protects them against proteolytic degradation after secretion [2]. It also modulates enzyme activity and cell recognition [3] [4] [5]. Additionally, the solubility of the protein in aqueous solvents is enhanced [6] [7]. The two types of glycosylation are N- and O-linked protein glycosylation which occur on asparagine and serine/threonine residues, respectively. N-glycans, highly conserved in evolution of all eukaryotic systems, including their diverse functions have been most extensively studied and are most common. N-linked glycosylation is involved in many cellular and biochemical processes, however not all functions are uncovered yet [8] [9] [10]. N-linked glycans seem to be essential for the activity of some enzymes such as α-amylase [11] or yeast α-amylase [12]. Both enzyme activities decreased and kinetic properties changed after removal of N-linked glycans by site-directed mutagenesis. Investigations concerning the requirement of glycans for stable conformation and full expression of the catalytic activity of an enzyme were also carried out by Lige et al. [13]. Using site-directed mutagenesis three different N-glycosylation sites were removed from cationic peanut peroxidase, one at the time. All three glycans appeared to influence protein folding and two of the three glycans were required for full expression of the catalytic activity of the enzyme.

CPO is naturally secreted by the filamentous fungus *Caldariomyces fumago* (synonym *Leptoxyphium fumago*). This versatile heme-containing enzyme with a molecular weight of 42 kDa exhibits peroxidase, catalase and cytochrome P450-like activities in addition to catalyzing halogenation reactions [14] [15]. Chloroperoxidase is an extensively glycosylated monomeric enzyme with both N- and O-linked glycosyl chains [16]. The enzyme exists in two isomeric forms A and B [17] [18]. While both isoenzymes possess the same amino acid sequence and the same specific activity, they differ in their carbohydrate composition, namely form A is more heavily glycosylated (carbohydrate constitutes 19% of the total molecular weight) [16] [19]. In total, form A contains three potential asparagine-linked, six threonine-linked and five serine-linked glycans.

Expressing recombinant active CPO in *E. coli* showed that glycosylation is not a mandatory requirement for refolding and activity of this enzyme [20]. Similarly, active CPO could be expressed in *A. niger* [21]. Although the recombinant enzyme was overglycosylated the excess of glycosyl groups did not have a major effect on enzyme properties.

Here we demonstrate for the first time the isolation of a *C. fumago* mutant secreting inactive non-N-glycosylated CPO lacking the heme group.

## Materials and Methods

### Media

The complex media used for precultures contained 200 g soft boiled potatoes l^−1^ and 30 g glucose l^−1^. Fructose minimal medium for main cultures was composed of 50 g fructose l^−1^, 2 g NaNO_3_ l^−1^, 2 g KH_2_PO_4_ l^−1^, 2 g KCl l^−1^, 1 g MgSO_4_^.^7 H_2_O l^−1^ and 0.02 g FeSO_4_^.^7 H_2_O l^−1^ [22]. The mineral salt solution was autoclaved separately from the sugar solution.

### Preparation of *C. fumago* Spheroblasts and UV Mutagenesis

Preparation of *C. fumago* spheroblasts was performed as described previously [23]. After resuspension of the spheroblasts in regeneration medium the suspension was plated on ∼75 large petri dishes (diameter of 13.5 cm) containing fructose minimal agar medium. The plates were subjected to UV irradiation (254 nm, 4 min, distance of 30 cm) using a Spectroline ENF-260C/FE lamp (Spectronics Corporation, Westbury, NY, US). During preliminary tests, these parameters were found to yield a survival rate of about 20%. After irradiation, the plates were immediately stored in the dark and incubated at 24°C. After seven days, a slightly greenish coloured compact growing mutant was isolated and transferred to a fructose minimal agar plate. Afterwards the mutant was cultivated on glucose potato agar plates and the mycelia were used to inoculate plates and liquid media for further experiments.

### Suspension Cultivation of *C. fumago*

Precultivation in 300 ml shake flasks containing 100 ml potato glucose medium was performed at 24°C and 160 rpm on a rotary shaker after inoculation with 1 cm^2^ *C. fumago* DSM 1256 mycelium from a glucose potato agar plate. For shake-flask cultivations in fructose minimal medium, 100 ml medium in 300 ml shake flasks were inoculated with 1 ml of a five to seven day old glucose potato medium preculture, that had been homogenized beforehand (Ultra-Turrax T25 basic; IKA Labortechnik; position III, 20 s). The fructose minimal cultures were incubated at 24°C on a rotary shaker at 200 rpm. One ml of a 1 g ml^−1^ fructose solution was added seven and nine days after inoculation of the cultures to prevent alkalinization of culture medium by *C. fumago.*

### Absorption Wavelength Scan

The absorption spectra of purified CPO were recorded with a UV-1700 Pharma Spectrophotometer (Shimadzu) over the range of 260–600 nm. CPO of wildtype and cpo1 was purified by aqueous biphasic systems as described later. Protein concentration was equalized by dilution of the samples with 0.1 M citric acid buffer solution (pH 4).

### Determination of CPO Activity

The CPO content of culture supernatants was determined by the monochlorodimedone (MCD) assay described by Morris and Hager [14] with slight modifications. One ml samples of the cultures were centrifuged (1 min, 16100 *g*) and appropriate dilutions of resulting supernatants were applied in a 100 mM citric acid buffer solution (pH 2.75) containing 0.1 M MCD and 2.3 mM H_2_O_2_ at 25°C and the decrease of absorbance at 278 nm was measured. To calculate enzyme activity a molar extinction coefficient for MCD of 12,200 M^−1 ^cm^−1^ [24] was used.

### SDS-PAGE Analysis

Protein separation was performed by discontinuous SDS-PAGE described by Laemmli [25]. Forty µL of supernatants from fructose minimal cultures were mixed with 10 µL of 5-fold concentrated SDS/2-mercaptoethanol-sample buffer (0.32 M Tris/HCl pH 6.8, 25% (w/v) ß-mercaptoethanol, 10% (w/v) SDS, 0.1% (w/v) bromophenol blue, 50% (v/v) glycerol). For the deglycosylation samples 8 µg protein of each deglycosylation reaction were mixed with an appropriate volume of 5-fold concentrated SDS/beta-mercaptoethanol-sample buffer, incubated at 95°C for 10 min and loaded on a 1.5 mm thick 15% polyacrylamide gel. Electrophoresis was carried out at a constant voltage of 90 V for 7 h at 4°C. The gel was subsequently stained with Coomassie Brilliant Blue G250 [26]. As a protein size marker the PageRuler™ Prestained Protein Ladder from Fermentas was used.

### CPO Gene Sequencing

The known CPO gene sequence including 926 nucleotides of promoter sequence and 720 nucleotides of terminator sequence was amplified from genomic DNA of wild type and cpo1 strains using the primers CPO-Hind5 (ACTGAAGCTTCTGGTTTGCATGCACTTGCATC) and CPO-Xba3 (ACTGTCTAGATGCCTTATACTAACACTCAGATCAG). The PCR fragments were cloned into pGEM-T vector (Promega) and sequenced afterwards.

### Purification of CPO Using Aqueous Biphasic Systems

CPO was concentrated for deglycosylation experiments using the protocol of Yazbik et al. [27] with slight modifications. To form the first biphasic system, 1.875 g PEG4000, 6.25 g PPG1200 and 0.5 g NaCl were dissolved in 16.25 ml culture supernatant of 10 or 11 day old fructose minimal medium cultures (final volume 23.75 ml). The solution was thoroughly mixed in a falcon tube and transferred to a separating funnel where phase separation occurred within about 15 min. After settling, the bottom phase (PEG phase) was collected and 2% (w/v) NaCl and 15% (w/v) (NH_4_)_2_SO_4_ were added to form a second biphasic system. After settling, CPO containing bottom phase (salt phase) was separated and buffer was exchanged by five-times 15 mL volumes of 250 mM sodium phosphate buffer through 10,000 MWCO membrane using Amicon Ultra centrifugal filter units (Millipore, Carrigtwohill, Co. Cork, Ireland).

### Deglycosylation of CPO

Twenty µg of glycoprotein purified by aqueous biphasic systems were used for deglycosylation experiments. Deglycosylation was performed with the Enzymatic CarboRelease™ Kit from QA-bio following the manufactureŕs instructions. *N*-linked oligosaccharides were removed using the enzyme PNGase F (*E. meningosepticum*), *O*-linked oligosaccharides were removed using the enzymes *O*-glycosidase (*S. pneumoniae*), sialidase (*A. ureafaciens*), ß-galactosidase (*S. pneumoniae*) and glucosaminidase (*S. pneumonia*). Fetuin was used as a positive control of the deglycosylation reactions and efficiency of deglycosylation was tested by running samples containing 8 µg protein on a SDS-PAGE gel.

### Protein Identification by Mass Spectrometry

For identification of the 38–40 kDa protein secreted by the mutant lacking CPO activity the protein band was excised from a Coomassie-stained SDS-PAGE gel. Protein spots were in-gel digested by trypsin and applied to nanoLC-ESI-MS/MS (Proteome Factory AG, Berlin, Germany). Proteins were identified using MS/MS ion search of the Mascot search engine (Matrix Science, London, England) and nr protein database (National Center for Biotechnology Information, Bethesda, USA).

## Results

**Figure 1:**
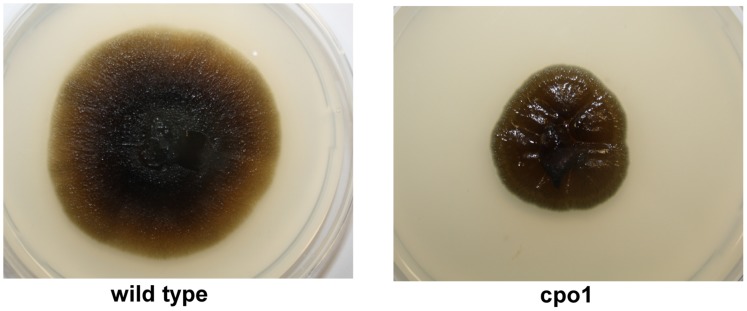
Colony color and growth of *C.* *fumago* wild type and cpo1 mutant on solid media. *C. fumago* wild type strain and the cpo1 mutant were plated on fructose minimal medium plates with a piece of mycelium grown on a glucose potato agar plate and incubated at 24°C for 7 days.

Spheroblasts of *C. fumago* were plated on fructose minimal medium and mutagenized by UV irradiation with a dose corresponding to a survival rate of 21%. Of about 26.000 single colonies that were visually inspected, one colony (cpo1) could be identified which was slightly greenish colored and grew slower in comparison to the wild type (Fig. 1). The mutant mycelium showed radial furrows and grew more compact on the agar plate.

### *C. fumago* Mutant Cpo1 Producing Non-active CPO

**Figure 2:**
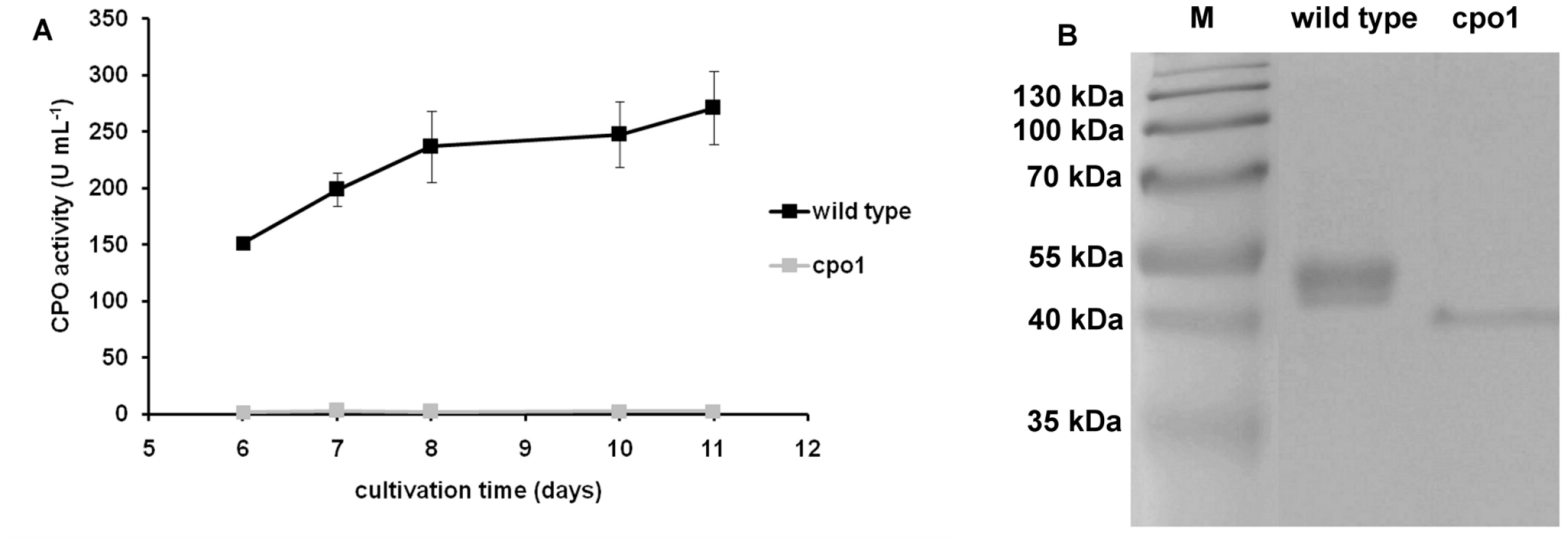
Comparison of CPO production characteristics of wild type and cpo1 strain. **A:** Comparison of CPO activities in supernatant samples of *C. fumago* wild type and cpo1 strain during cultivation in fructose minimal medium. Strain cultivation and determination of enzyme activity via the MCD assay was performed as described in the materials and methods section. Data is expressed as mean of values from two shake flasks ± standard deviation. **B:** Analysis of CPO protein levels in supernatant samples of *C. fumago* wild type and cpo1 strain by SDS-PAGE. SDS-PAGE analysis was performed as described in the materials and methods section. M: molecular weight marker. The numbers on the left indicate the sizes of the molecular weight marker.

Analysis of the culture supernatants of fructose minimal medium cultures showed that only low CPO activity (a maximum of 3.2 U mL^−1^) could be detected for cpo1 (Fig. 2 A). Protein analysis of supernatant samples via SDS-PAGE also revealed the absence of the CPO band at around 42 kDa for the mutant sample. Interestingly, an additional band at 38–40 kDa appeared on the gel which could not be detected in the wild type supernatant (Fig. 2 B). The fact that this band was not present in a sample from a glucose medium culture (data not shown) provided some evidence for the 38–40 kDa protein being CPO as production of this enzyme is repressed by glucose and induced by fructose in *C. fumago* [28]. To confirm this hypothesis the protein band was excised from the SDS-PAGE gel and protein identification using nanoLC-ESI-MS/MS was performed (Proteome Factory AG, Berlin, Germany). Three peptides QGVANSNDFIDNR (score 84), THFDYADMNEIR (score 72) and NFDAETFQTSLDVVAGK (score 107) matching CPO were identified. Sequencing of wild type and cpo1 CPO gene showed that both gene sequences are identical.

### Lack of the Heme Group in the CPO Variant Secreted by the Cpo1 Mutant

**Figure 3:**
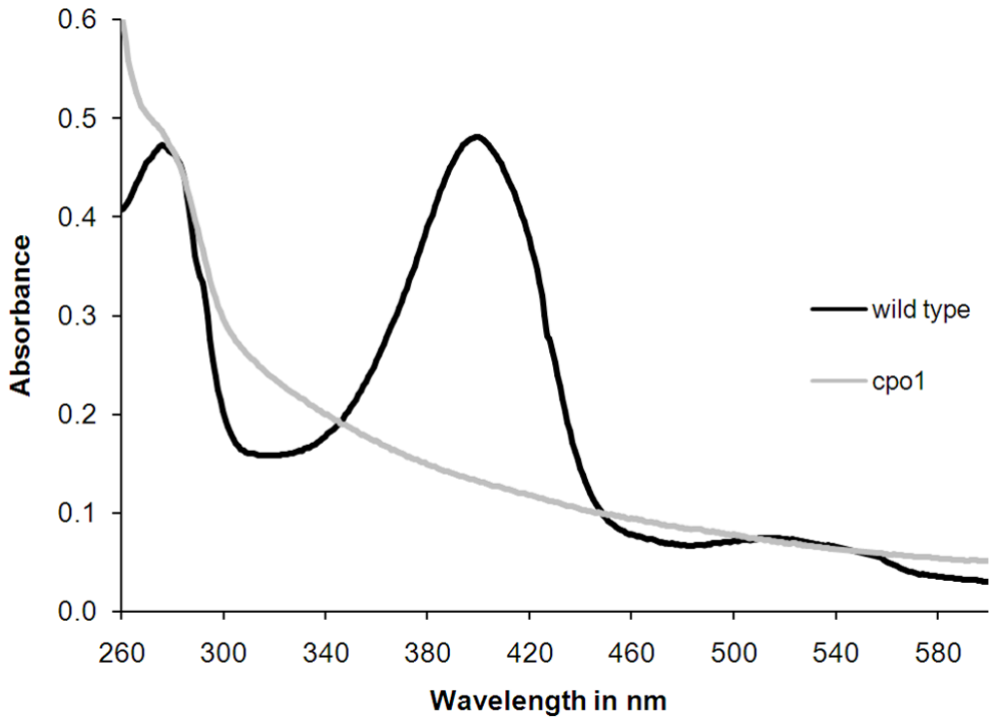
UV-Vis spectra of *C.* *fumago* wild type and cpo1 CPO purified by aqueous biphasic systems. Culture supernatants from fructose minimal medium cultures grown for 11 days on a rotary shaker at 24°C each were purified using aqueous biphasic systems and protein concentration was equalized by dilution of the samples with 0.1 M citric acid buffer solution (pH 4). CPO activity of the diluted samples was determined as 246.46 U mL^−1^ and 0.03 U mL^−1^ for wild type and cpo1, respectively.

Wavelength scans of *C. fumago* wild type and cpo1 CPO purified by aqueous biphasic systems were performed in order to get more information about the spectral properties of the culture supernatant constituents. The Soret absorbance band typical for the heme group of CPO in the 400 nm spectral region as well as the beta-band at 530 nm could be detected for the wild type sample (figure 3). The spectrum of the cpo1 sample lacked both characteristic bands. The ratio between A_400_ (indicating heme-containing protein) and A_280_ (indicating total protein), R_Z_ value, is lower for cpo1 (0.28) in comparison to *C. fumago* wild type (1.03). Pure CPO has an R_Z_ of 1.44 [29] suggesting that CPO of the cpo1 strain is not occupied with heme.

### CPO of Cpo1 Mutant Lacks *N*-glycosylation

**Figure 4:**
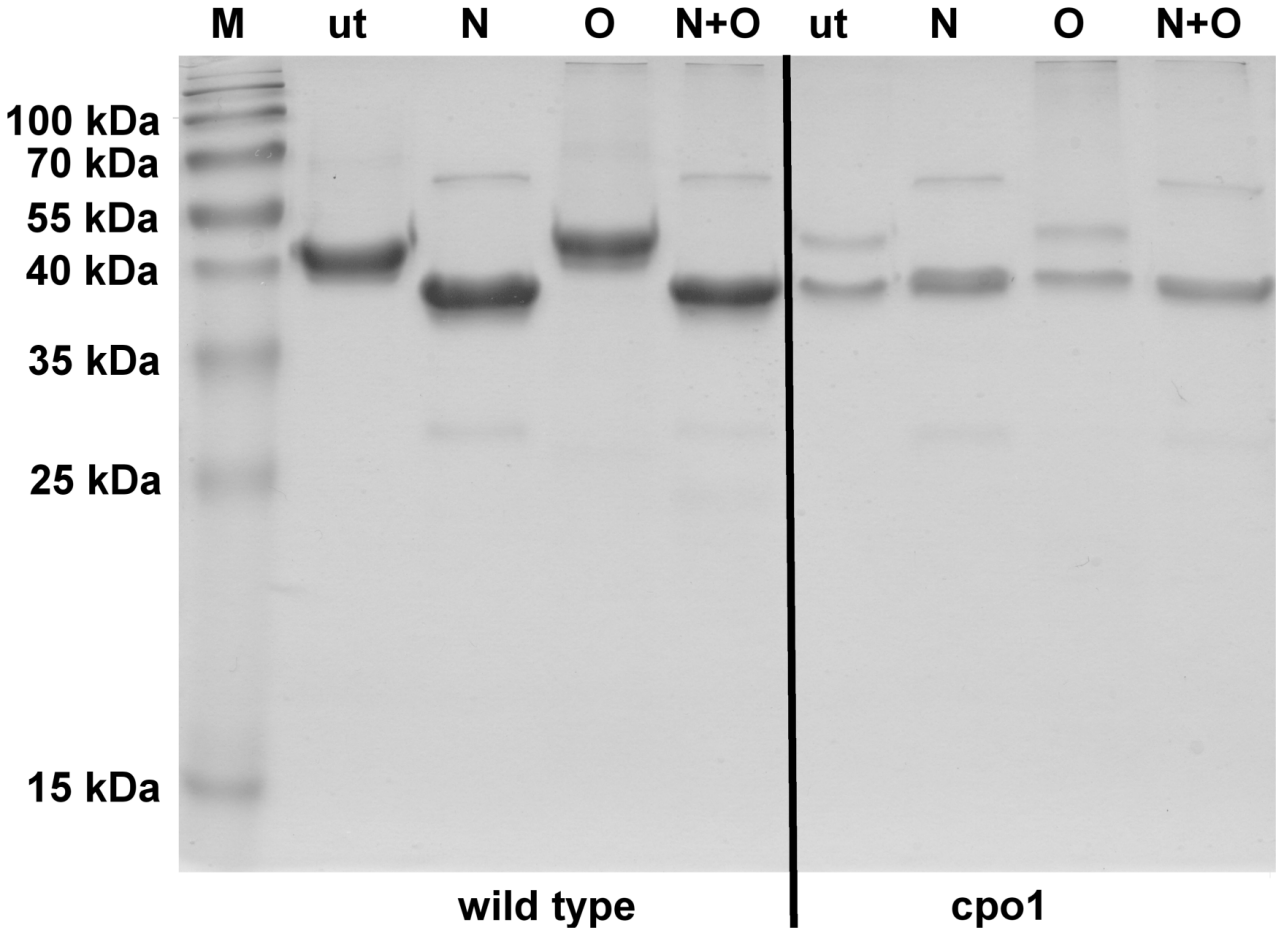
SDS-PAGE analysis of CPO protein sizes after deglycosylation. Culture supernatants from fructose minimal medium cultures grown for 10 days on a rotary shaker at 24°C each were purified using aqueous biphasic systems and deglycosylation was performed as described in the materials and methods section. N+O: glycosidases were added to remove N- and O-linked oligosaccharides, O: glycosidases were added to remove O-linked oligosaccharides, N: glycosidases were added to remove N-linked oligosaccharides, ut: untreated sample, M: molecular weight marker. The numbers on the left indicate the sizes of the molecular weight marker.

The results shown in figure 2 B led to the hypothesis that a glycosylation defect in the cpo1 strain is responsible for the appearance of a 4 kDa smaller protein. Therefore deglycosylation experiments with purified CPO samples from wild type and cpo1 were performed. The cpo1 culture supernatant used for this experiment contained small amounts of the 42 kDa protein in addition to the 38–40 kDa protein. Twenty µg purified CPO from wild type and cpo1 were mixed with glycosidases to remove either *N*-linked, either *O*-linked or *N*- and *O*-linked oligosaccharides from the glycoprotein. Weak bands greater than 55 kDa and smaller than 35 kDa appearing in the SDS-PAGE gel can be attributed to the different glycosidases used. Detected CPO activity of untreated sample was 2.6 U mL^−1^. Whereas all cpo1 samples showed no significant change in protein size of the 38–40 kDa protein after treatment with the different glycosidases wild type CPO differed in protein size after deglycosylation (Fig. 4). The removal of all *O*-linked oligosaccharides did not cause a visible shift of the protein band of wild type CPO but treatment with enzymes which either N- or N- and O-deglycosylated the CPO led to a decrease in protein size. Thus, the molecular mass of N-deglycosylated CPO from wild type is 38–40 kDa. As the untreated cpo1 sample did also show a protein band at the same size it can therefore be concluded that the 38–40 kDa protein secreted by the cpo1 strain lacks at least the *N*-glycans.

### Phenotypic Instability of *C. fumago* Mutant Cpo1

**Figure 5:**
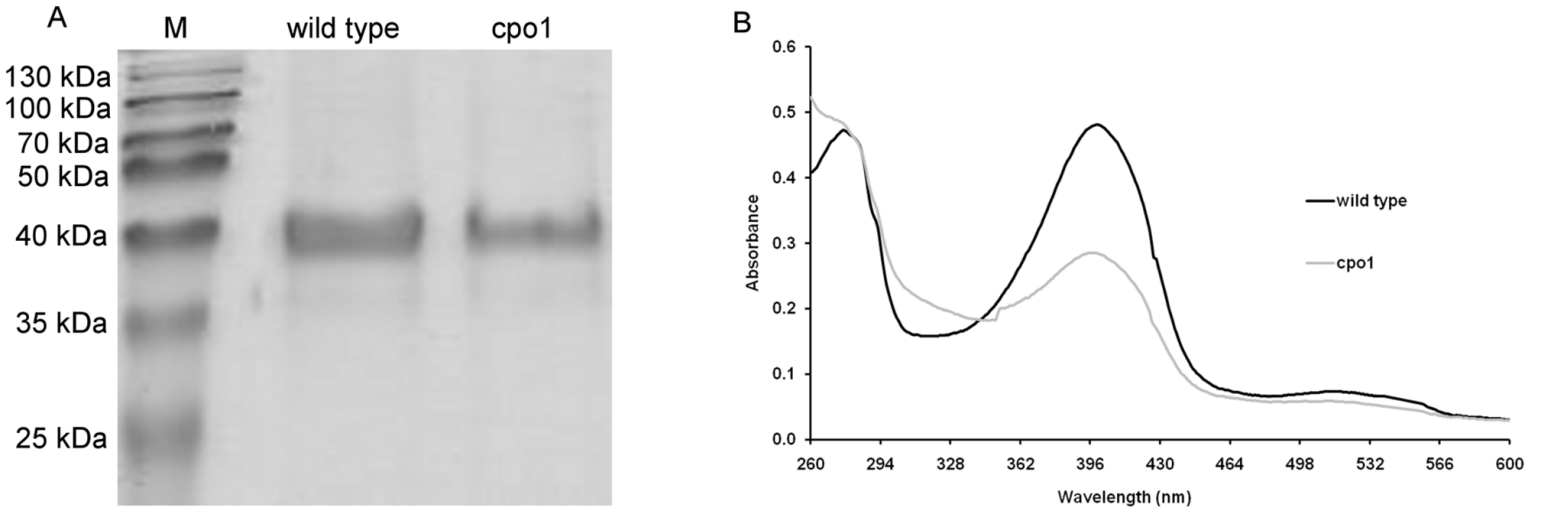
Instability of cpo1 strain. **A:** Analysis of CPO protein levels in supernatant samples of *C. fumago* wild type and cpo1 strain by SDS-PAGE. SDS-PAGE analysis was performed as described in the materials and methods section. The numbers on the left indicate the sizes of the molecular weight marker. M: molecular weight marker. CPO activity of fructose minimal medium cultures of wild type and cpo1 was determined as 60 U mL^−1^ and 38 U mL^−1^, respectively. **B:** UV spectra of *C. fumago* wild type and cpo1 CPO purified by aqueous biphasic systems. Culture supernatants from fructose minimal medium cultures grown for 11 days on a rotary shaker at 24°C each were purified using aqueous biphasic systems and protein concentration was equalized by dilution of the samples with 0.1 M citric acid buffer solution (pH 4).

As already shown in figure 4, two bands appeared on the SDS-PAGE gel of the untreated cpo1 sample (38–40 kDa and 42 kDa), but the sample showed neglectable CPO activity. Further independent experiments with the cpo1 strain showed that during some cultivations either only the 38–40 kDa protein, both the 42 kDa and the 38–40 kDa protein or even only the 42 kDa protein appeared. The latter case is presented in figure 5 A. The respective cpo1 sample furthermore exhibited not only the Soret band demonstrating heme presence (figure 5 B) but also significant CPO activity (38 U ml^−1^). Different cultivation times of the preculture (5 or 7 days) as well as different ages of the strain maintenance plate could be excluded as possible causes for the variable behavior of the mutant strain.

## Discussion

Here we demonstrate the isolation of a *C. fumago* mutant defective in CPO production by visual screening of colonies from randomly mutagenized spheroblasts. Usually no or at least a considerably decreased CPO activity was detected in the supernatant sample of the mutant strain. SDS-PAGE analysis of cpo1 culture supernatant proteins revealed that the CPO band disappeared almost completely and an additional band at a lower size of 38–40 kDa appeared. Since this protein could be identified as CPO by peptide mass fingerprinting and the gene sequences of CPO from wild type and cpo1 mutant were identical we assumed the glycosylation of CPO to be affected. N-deglycosylation experiments using purified CPO from wild type and cpo1 mutant followed by SDS-PAGE analysis proved that for the wild type strain the native CPO band disappeared and N-deglycosylated CPO was resolved into a major band of about 38–40 kDa. Therefore we conclude that indeed at least N-glycosylation of CPO is affected in the mutant.

Expression of recombinant CPO in *E. coli* showed that glycosylation is not a mandatory requirement for the refolding and activity of the non-native enzyme [20]. When expressing CPO in *A. niger* even excess of glycosyl groups did not have a major effect on the properties of the recombinant enzyme [21].

Haines [30] already performed *in vitro* deglycosylation experiments with non-denatured active CPO cleaving the glycosidic bonds of asparagine-linked carbohydrates. SDS-PAGE analysis of the deglycosylation reaction sample also revealed the disappearance of the native CPO band being replaced by a protein band of 37.5 kDa. The *in vitro* N-deglycosylated CPO yielded 75% of the activity of native CPO. DNA sequencing predicted a molecular weight of around 38.2 kDa for the nonglycosylated polypeptide [24]. Haineś results show that once the enzyme is properly folded and secreted subsequent N-deglycosylation does not affect enzyme activity. Concluding, the lack of N-glycans of the 38–40 kDa protein secreted by the cpo1 strain most probably is not the cause for the loss of enzyme activity. Furthermore N-glycosylation seems to be no prerequisite for secretion of this enzyme.

As can be seen in figure 2, the spectrum of the mutant supernatant sample lacked the Soret band as well as the beta-band typical for the heme group of CPO demonstrating the absence of heme in the protein secreted by the mutant. As the heme is essential for the catalytic property its lack in the CPO variant is without doubt the causal reason for the observed absence of enzyme activity.

As shown in figure 5 the mutant strain did in some cultivations produce the correctly glycosylated and heme-containing active protein to some extent. Further research will be necessary to uncover the mechanism behind this instability phenomenon.

Up to now no information exists about the time course of heme incorporation and glycosylation of CPO in *C. fumago*. Several factors are involved in the biogenesis of secreted glycosylated heme enzymes. In order to achieve their final structure, proteins are subjected to a complex series of processing events including polypeptide folding, transient interactions with molecular chaperones, protease cleavage reactions, glycosylation and heme incorporation [31], [32]. To our knowledge only one secreted heme-containing protein has been investigated intensively with regard to its biogenesis pathway. Hansson et al. [31] reviewed the data available about human myeloperoxidase synthesis, where N-glycosylation occurs before incorporation of covalently bound heme. For the same protein Nausseef et al. [33] proposed heme insertion to be a prerequisite for further proteolytic processing to the mature active enzyme. From the data shown here for CPO it is currently not possible to conclude without doubt that a causal link exists between heme insertion and glycosylation. Identification of the mutation responsible for the defects of the cpo1 strain and analysis of possible causal connections between the different effects are necessary to draw a more detailed picture of the CPO maturation pathway. The primary defect might e.g. concern proteolytic processing or correct folding of the enzyme. Gaining more insights into the essential CPO maturation events may open the possibility for recombinant production of this valuable enzyme in the future.
